# Occupational Bladder Cancer in a 4,4′-Methylenebis(2-chloroaniline) (MBOCA)-Exposed Worker

**DOI:** 10.1289/ehp.7666

**Published:** 2005-02-25

**Authors:** Chiu-Shong Liu, Saou-Hsing Liou, Ching-Hui Loh, Yi-Chun Yu, Shi-Nian Uang, Tung-Sheng Shih, Hong-I Chen

**Affiliations:** ^1^Department of Family Medicine, China Medical University, Taichung, Taiwan, Republic of China;; ^2^Department of Public Health, National Defense Medical Center, Nei-Hu, Taipei, Taiwan, Republic of China;; ^3^Division of Environmental Health and Occupational Medicine, National Health Research Institutes, Kaohsiung, Taiwan, Republic of China;; ^4^Department of Family Medicine and Internal Medicine, Tri-Service General Hospital, National Defense Medical Center, Nei-Hu, Taipei, Taiwan, Republic of China;; ^5^Institute of Occupational Safety and Health, Council of Labor Affairs, Shi-Jr, Taipei, Taiwan, Republic of China;; ^6^Department of Surgery, Tri-Service General Hospital, National Defense Medical Center, Nei-Hu, Taipei, Taiwan, Republic of China

**Keywords:** bladder cancer, MBOCA, transitional cell carcinoma

## Abstract

A 52-year-old male chemical worker was admitted to the hospital with a history of paroxysmal microscopic hematuria for about 2 years and nocturia with gross hematuria about five times per night for 2 months. He was a nonsmoker and denied a history of any other bladder carcinogen exposure except for occasional pesticide application during agricultural work. Intravenous urogram imaging showed a mass occupying half of the bladder capacity. Cystoscopy revealed a mass over the left dome of the bladder. Cystoscopic biopsy revealed a grade 3 invasive transitional cell carcinoma with marked necrosis. From 1987 until hospital admission in 2001, the patient had worked in a company that produced the 4,4′-methylenebis(2-chloroaniline) (MBOCA) curing agent. He did not wear any personal protective equipment during work. Ambient air MBOCA levels in the purification process area (0.23–0.41 mg/m^3^) exceeded the U.S. Occupational Safety and Health Administration’s permissible exposure level. Urinary MBOCA levels (267.9–15701.1 μg/g creatinine) far exceeded the California Occupational Safety and Health Administration’s reference value of 100 μg/L. This patient worked in the purification process with occupational exposure to MBOCA for 14 years. According to the environmental and biologic monitoring data and latency period, and excluding other potential bladder carcinogen exposure, this worker was diagnosed as having occupational bladder cancer due to high exposure to MBOCA through inhalation or dermal absorption in the purification area. This case finding supports that MBOCA is a potential human carcinogen. Safe use of skin-protective equipment and respirators is required to prevent workers from MBOCA exposure.

Incidence and mortality rates of bladder cancer vary about 10-fold worldwide. The highest rates are found in North America and Europe, and the rates are low in many parts of Asia (Engel et al. 2002). In Taiwan, the incidence and mortality rates of bladder cancer (per 100,000) in 2000 were 8.93 and 3.00 for males and 3.87 and 1.36 for females, respectively [Taiwan Department of Health (DOH) 2004]. Transitional cell carcinomas account for about 95% of bladder neoplasms. The remaining 5% consist of squamous cell carcinomas, adenocarcinomas, and others (Engel et al. 2002). Cigarette smoking and occupational exposures are well-documented risk factors for bladder cancer. Other known or suspected risk factors for bladder cancer include race, sex, age, lifestyle, chlorination by-products and arsenic in drinking water, ionizing radiation, bladder infection, high consumption of phenacetin-containing analgesics, and hair dyes (Engel et al. 2002). Several genetic susceptibility factors have been found to be related to bladder cancer (Engel et al. 2002).

Bladder cancer is associated with a number of occupational exposures. The first such association was observed in 1895 (Rehn 1895), and subsequent research among dyestuff workers identified the aromatic amines benzidine and 2-naphthylamine as bladder carcinogens (Case et al. 1954). Several other aromatic amines and related compounds have also been identified as suspected human bladder carcinogens, including 1-naphthylamine, 4-aminobiphenyl, 4-chloro-*o*-toluidine, *o*-toluidine, and 4,4′-methylenedianiline [Engel et al. 2002; International Agency for Research on Cancer (IARC) 1987]. Excess risk of bladder cancer has also been observed among rubber workers; painters; truck, bus, and taxi drivers; aluminum workers; and leather workers (Engel et al. 2002). It has been estimated that these occupational exposures are responsible for 18% of bladder cancer cases. As little as 2 years’ exposure may be sufficient to increase the risk, but the time between exposure and subsequent cancer may be as long as 45 years (Goroll et al. 2000).

4,4′-Methylenebis(2-chloroaniline) (MBOCA) is used as a curing agent in industries that primarily produce castable polyurethane parts; thus, occupational exposure may occur during the manufacturing processes in these industries [Agency for Toxic Substances and Disease Registry (ATSDR) 1994]. Workers may inhale MBOCA in small particles in the air or absorb it through the skin during contact with MBOCA dust or vapor. Acute exposure to high levels of MBOCA may cause eye and skin irritation in humans (Hosein and Van Roosmalen 1978). Intermediate and chronic exposure to MBOCA may lead to tumors of the urinary bladder (ATSDR 1994; Kommineni et al. 1979; Russfield et al. 1975; Stula et al. 1975, 1978). In a U.S. retrospective bladder cancer incidence study, 385 of 532 workers ever exposed to MBOCA from 1968 to 1979 and 20 workers who were first employed in 1980 and 1981 participated in a urine screening test (Ward et al. 1990). Workers were exposed to MBOCA for a median employment period of 3.2 months (between 1968 and 1981). Cystoscopy revealed a papillary cell tumor in one worker, and low-grade papillary transitional cell tumors of the urinary bladder were diagnosed in 2 of the remaining 200 workers examined by cystoscopy (Ward et al. 1990). The U.S. Department of Health and Human Services has determined that MBOCA may reasonably be anticipated to be a carcinogen (ATSDR 1994). IARC has determined that MBOCA is probably carcinogenic to humans (category 2A; IARC 1993). The U.S. Environmental Protection Agency has determined that MBOCA is a probable human carcinogen (category 2A; ATSDR 1994). In the *Report on Carcinogens, Eleventh Edition* [National Toxicology Program (NTP) 2005], the NTP reported that MBOCA may reasonably be anticipated to be a human carcinogen. In this article we report a sentinel case of transitional cell carcinoma of the urinary bladder diagnosed in an MBOCA-manufacturing factory in Taiwan.

## Case Presentation

### Case report.

A 52-year-old male chemical worker was admitted to the hospital with a history of paroxysmal microscopic hematuria for about 2 years and nocturia with gross hematuria about five times per night for 2 months. He was a nonsmoker and denied taking any medication. He did not use hair dye or reside in an area with endemic blackfoot disease (arsenic intoxication). Social alcohol drinking was noted.

Microscopic hematuria was noted in the periodic health examinations for about 2 years, but the patient ignored it. Two months before admission, he developed nocturia about five times per night. Paroxysmal painless hematuria was also noted. Gross hematuria accompanied by lower abdominal distress occurred 2 weeks before admission. His body weight had decreased from 75 kg to 72 kg over the previous 3 months. He visited the hospital, and an intravenous urogram (IVU) showed a mass occupying half of the bladder capacity. Cystoscopy revealed a mass over the left dome area of the bladder. Cystoscopic biopsy specimens revealed a grade 3/3 invasive transitional cell carcinoma with marked necrosis. Radical cystectomy with ileal conduit combined with radiotherapy was performed because the bladder tissue showed lymphovascular permeation with lymph node metastasis. The worker is still on medical leave because of disease.

### Occupational history.

The manufacturing of MBOCA includes the reaction, neutralization, washing, purification, and packing processes. Briefly, *o*-chloroaniline is reacted with sulfuric acid and formaldehyde under the catalyst of stannous chloride for 3–4 hr. The MBOCA products are then neutralized with an alkali, sodium hydroxide, at a high temperature. After washing and purification, the solid MBOCA is cut into pellets. Although most of the processes are done in closed systems and are automatic, leakage of products from pipes and tanks has been reported. The work schedule in the factory where our patient worked has three 8-hr shifts, and three or four workers in each shift are assigned to each manufacturing line.

The patient was a farmer until he began working at this factory. He worked in the purification area of this MBOCA-producing company for 14 years (1987–2001), and he did not wear any personal protective equipment during work. He denied working for any chemical company other than his present company. He occasionally applied pesticides during agricultural work.

### Environmental monitoring data.

An area sampling program to monitor MBOCA levels in the work environment of this MBOCA manufacturing factory was initiated by the Taiwan Institute of Occupational Safety and Health (IOSH), Council of Labor Affairs (IOSH 2003a). The U.S. Occupational Safety and Health Administration’s (OSHA) Analytic Method 24 was adopted in this study (OSHA 1981). Briefly, an impinger filled with 0.1N HCl was used for sampling. The sampling rate was 1 L/min. The sampling time was > 6 hr or > 100 L air. The solution was then analyzed with high-performance liquid chromatography (HPLC) with a 254 nm ultraviolet (UV) detector. The detection limit was 0.056 μg/mL. A quality assurance and quality control program was implemented during the sampling and analysis procedures. All quality tests were shown to be adequate (IOSH 2003a).

Two consecutive days’ air samples were collected in the MBOCA-manufacturing factory, in the areas where the reaction, neutralization, washing, purification, and cutting/packing processes took place. The concentration of MBOCA was highest in the purification area (0.23–0.41 mg/m^3^, *n* = 2), followed by the washing area (< 0.02–0.08 mg/m^3^, *n* = 7) and the neutralization area (< 0.05–0.06 mg/m^3^, *n* = 2) (IOSH 2003a). The concentrations of MBOCA were within OSHA permissible exposure level (0.22 mg/m^3^) except for the purification area, where levels exceeded permissible levels.

### Biological monitoring data.

In addition to environmental monitoring, the Taiwan IOSH also collected workers’ urine to monitor MBOCA concentrations. The U.S. National Institute for Occupational Safety and Health (NIOSH) analytic method 8302 (NIOSH 1994) and the method used by Robert et al. (1999) were adopted in this study. Briefly, sulfamic acid (10 g/L) was used as urine preservative, and urine was neutralized by sodium hydroxide (0.05 g/mL). After cleaning with the Extrelut NT3 column (Merck, Darmstadt, Germany) and evaporating with methanol, the concentrated urine was analyzed by HPLC with a 254 nm UV detector. The limit of detection was 9.54 μg/L.

Urine from 10 workers in this MBOCA-manufacturing company was analyzed. The total urine MBOCA concentrations ranged from 267.9 to 15701.1 μg/g creatinine, with a mean of 5,544 μg/g creatinine (IOSH 2003b). All the urine MBOCA concentrations far exceeded the California Occupational Safety and Health Administration (Cal-OSHA) reference value of 100 μg/L (Robert et al. 1999).

## Discussion

Although the production of MBOCA in the United States ceased in 1982, MBOCA is still manufactured in other countries. It is also widely used, primarily in industries producing castable polyurethane elastomers (ATSDR 1994). Therefore, the health impact of MBOCA is still of concern in occupational settings in many countries. In this article we report the first known case of MBOCA-related occupational bladder cancer in Taiwan, which led to the establishment of recommended exposure levels of MBOCA in the workplace. This case report supports the conclusion that MBOCA is a potential human carcinogen.

Environmental monitoring of MBOCA levels in ambient air performed in the present study showed that the concentration was high in the purification process area (0.23–0.41 mg/m^3^) and exceeded the OSHA permissible exposure level (0.22 mg/m^3^) and NIOSH’s recommended exposure level of MBOCA (3 mg/m^3^ as a 10-hr time-weighted average) (Ward et al. 1990). The production quantity of MBOCA at this patient’s plant was about 1,500 tons/year, which was much higher than the 184–580 tons/year reported by Ward et al. (1990). The air levels of MBOCA in this plant were also higher than those in a polyurethane elastomer factory using MBOCA as a curing agent (Clapp et al. 1991). No airborne MBOCA was detected in another polyurethane elastomer factory (Clapp et al. 1991). In another study, personal sampling of the breathing zone of workers for 6–7 hr every other day found levels ranging from 0.002 to 0.0089 mg/m^3^ (Ichikawa et al. 1990).

In addition to exposure to high air concentrations of MBOCA, workers in the patient’s plant also showed high levels of MBOCA in urine. The urine MBOCA concentrations ranged from 267.9 to 15701.1 μg/g creatinine with a mean of 5,544 μg/g creatinine. The urine MBOCA concentrations of all 10 workers far exceeded the Cal-OSHA reference value of 100 μg/L (Robert et al. 1999). Urine samples obtained by Ward et al. (1990) from plant workers several months after production ceased also showed detectable MBOCA levels that ranged as high as 50,000 μg/L. Osorio et al. (1990) reported a urine MBOCA level of 1,700 μg/L 4 hr postexposure in a worker who was accidentally sprayed with molten MBOCA, but no acute symptoms or other laboratory abnormalities were observed. The urine MBOCA levels in the MBOCA-manufacturing factory workers were much higher than in workers at a polyurethane elastomer factory using MBOCA as a curing agent. Clapp et al. (1991) reported concentrations for mixers in the polyurethane elastomer factory ranging from 5 μg/L to > 100 μg/L urine (average of 61.9 μg/L), whereas concentrations for molders were considerably lower (nondetectable to 50 μg/L urine; average of 14.8 μg/L). Workers in a plant that used MBOCA had urine concentrations of MBOCA ranging from 13 to 458 μg/L (mean 145 μg/L; Keeslar 1986). Another study of 150 workers with industrial exposure to MBOCA in 19 factories showed that, at the end of the work shift, excretion levels of MBOCA ranged from < 0.5 μg/L to 1,600 μg/L, with the highest average urine concentrations (600 μg/L) in workers directly involved in MBOCA manufacture or use (Ducos et al. 1985). In a NIOSH study of urine samples from mixers and molders in a polyurethane elastomer factory (NIOSH 1986), the average urine MBOCA level during the week was approximately 30 μg/L for a mixer over a 5-day work week; the level dropped to 8.9 μg/L over the weekend. However, in Australia, monitoring of workers at seven facilities that used MBOCA in the manufacture of polyurethane polymers showed that the average MBOCA levels in the urine of the workers dropped from 29,600 to 10,400 μg/L within 8–9 months after the implementation of an exposure prevention program (Wan et al. 1989).

The bladder cancer patient reported here worked in the purification process with high exposure to MBOCA for 14 years. He did not wear any personal protective equipment during work. Workers may inhale small particles of MBOCA in the air or absorb the agent through the skin if they come into contact with MBOCA dust or vapor (Chin et al. 1983; Cocker et al. 1988, 1990; Ichikawa et al. 1990; NIOSH 1986). According to the environmental monitoring data for the patient’s factory, he may have been exposed to high concentrations of MBOCA through inhalation or dermal absorption.

Limited epidemiologic studies have examined the incidence of cancer in workers exposed to MBOCA, and three cases of bladder cancer have been reported to be associated with MBOCA exposure. Ward et al. (1990) screened 385 MBOCA-exposed workers and revealed a papillary tumor in one worker after cystoscopy; two low-grade papillary transitional cell carcinomas of the urinary bladder were diagnosed in two of the remaining 200 workers examined by cystoscopy. Two of the men with bladder cancer in Ward et al.’s study (Ward et al. 1988) were younger than 30 years of age. The interval between the time of first exposure and the initiation in that study averaged 11.5 years. The latency period in our case was 14 years, which was compatible with that reported by Ward et al. (1988). The present study adds evidence to the potential carcinogenicity of MBOCA. Human epidemiologic findings are supported by results obtained in dogs (Stula et al. 1978). Results in other animal species also support the conclusion that MBOCA is a potential carcinogen (ATSDR 1994). In addition to the bladder, other target organs include the lung, liver, breast, and Zymbal’s gland in rats (Kommineni et al. 1979; Russfield et al. 1975; Stula et al. 1975) and the lung, liver, and vascular system in mice (Russfield et al. 1975). No adequate epidemiologic studies of MBOCA had been conducted. The present case supports the conclusions from other studies that MBOCA is potentially carcinogenic to humans as well as animals, but it does not substitute for the gap between epidemiologic evidence and animal studies.

## Conclusion

This patient was a nonsmoker. Because no other potential risk factors of bladder cancer could be found, occupation-related cancer was strongly suspected. This case finding supports the conclusion that MBOCA is a potential human carcinogen. In most cases, dermal absorption is the most important occupational exposure pathway. Workplace monitoring by air sampling has been reported to be useless because contamination may occur in the absence of observable air levels (Clapp et al. 1991). Biological monitoring of urine MBOCA concentration is a useful method to assess percutaneous MBOCA absorption. Because MBOCA is a potential human carcinogen, it is important to reduce exposure primarily through engineering ventilation controls. However, safe use of skin protective equipment and respirators is required to prevent MBOCA exposure. In addition, appropriate clearance of spills, work training, air monitoring, and periodic health examinations are recommended (ATSDR 1994; Clapp et al. 1991).

## Figures and Tables

**Figure:**
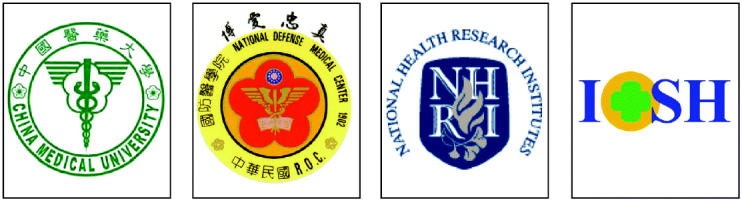
China Medical University, National Defense Medical Center, National Health Research Institutes, Institute of Occupational Safety and Health
